# Targeted long-read sequencing for high-resolution repeat profiling in myotonic dystrophy type 1

**DOI:** 10.1038/s12276-026-01683-6

**Published:** 2026-04-13

**Authors:** Yoojung Han, Ja-Hyun Jang, Hyeshik Chang

**Affiliations:** 1 Center for RNA Research, Institute for Basic Science, Seoul, Republic of Korea; 2 Interdisciplinary Program in Bioinformatics, Seoul National University, Seoul, Republic of Korea; 3 Department of Laboratory Medicine and Genetics, Samsung Medical Center, Sungkyunkwan University School of Medicine, Seoul, Republic of Korea; 4 School of Biological Sciences, Seoul National University, Seoul, Republic of Korea

**Keywords:** Targeted resequencing, Computational platforms and environments, Genetic testing, Genetic testing

## Abstract

Tandem repeat expansion disorders can be difficult to diagnose when expansions exceed 200 repeats, as standard methods (for example, Southern blot and modified PCR) often fail. We present a Cas9-targeted nanopore sequencing workflow and an automated analysis pipeline, RepeatLab, for accurate repeat-length estimation, structure assessment, and high-resolution methylation profiling. Validated on 13 myotonic dystrophy type 1 samples, 4 healthy controls, and 4 cell lines, this approach demonstrates improved sensitivity and accuracy for large expansions. Key refinements include an alternative basecalling strategy for extended repeats and a repeat-length calling algorithm that remains robust at lower sequencing throughput. The platform also automatically reports methylation near the *DMPK* repeat region, including five CpG site groups that could inform more nuanced clinical evaluations. This integrated workflow offers a rapid, cost-effective diagnostic solution with a turnaround time under 24 h and costs comparable to standard assays. Its compatibility with readily available computational resources enhances accessibility and scalability.

## New workflow streamlines diagnosis of myotonic dystrophy type 1

Huntington’s disease, myotonic dystrophy, and fragile X syndrome are disorders caused by abnormal DNA sequences called repeat expansions. These expansions lead to various symptoms, including muscle weakness and cognitive issues. Diagnosing these disorders accurately is challenging owing to the technical difficulties of measuring repeat sizes. Researchers developed a new diagnostic platform called RepeatLab that combines Cas9-enriched nanopore sequencing and an automated analysis pipeline. The researchers tested their method on samples from 13 patients and 4 controls. Their results showed that RepeatLab provides both accurate repeat-length measurements and DNA methylation patterns at single-molecular resolution within a single workflow. RepeatLab improves the diagnosis of repeat expansion disorders and helps understand their underlying mechanisms. Future work may focus on refining this method for broader clinical use.

This summary was initially drafted using artificial intelligence, then revised and fact-checked by the author.

## Introduction

Huntington’s disease, myotonic dystrophy, and fragile X syndrome are neurodegenerative disorders characterized by abnormal expansions of short tandem repeats^1–3^. These expansions cause a diverse range of symptoms, including cognitive and physical deficits as well as psychological and behavioral issues. Myotonic dystrophy type 1 (DM1) is a prime example, arising from an expanded triplet repeat sequence in the 3′ untranslated region (UTR) of the *DMPK* gene^4,5^. With a prevalence of approximately 1 in 2100 people^6^, DM1 presents with symptoms such as muscle weakness, fatigue, and balance problems^7^. The severity of the disease is correlated with the length of the repeat expansion, highlighting the importance of accurate repeat size estimation for effective diagnosis^8–12^.

Diagnosis methods for repeat expansion disorders vary based on the range of full penetrance alleles^13–15^. Fluorescent fragment analysis with flanking primers of the region of interest (ROI) is effective in detecting a small number of pathogenic repeats. For larger repeat numbers, Southern blot can address the inefficient amplification of alleles with extensive repeats, despite being time-consuming and labor-intensive^16,17^. Repeat primed PCR (RP-PCR), an alternative to Southern blot, is widely used and uses a primer targeting the repeated sequence along with fluorescent primers targeting flanking sequences^18–22^. Nevertheless, establishing this assay can be challenging owing to the GC-rich nature of repeated sequences, which complicates the identification of optimal conditions^23^.

Long-read sequencing has emerged as a promising alternative to address the limitations of existing approaches^24–35^. Among these platforms, nanopore DNA sequencing by Oxford Nanopore Technologies (ONT) offers rapid turnaround time, a streamlined workflow, and substantially lower equipment costs. However, for routine clinical applications, further advancements in cost-effectiveness are required, which could be achieved through targeted enrichment strategies. PCR-based target amplification, one such strategy, is ill-suited for regions with repeat expansions, as larger expanded alleles may not amplify effectively. Hybridization capture suffers from inadequate targeting specificity for probes targeting repetitive sequences. Recently, Cas9-targeted enrichment has been explored for the diagnosis of repeat expansion disorders^27,36–41^. In this approach, an ribonucleoprotein (RNP) complex comprising guide RNA and Cas9 recognizes and cleaves the ROI, followed by adapter ligation, enabling specific gene target enrichment^42^.

Several tools, including RepeatHMM^43^, NanoRepeat^44^, DeepRepeat^45^, and STRique^36^, have been developed to estimate repeat lengths from nanopore sequencing data, with RepeatHMM and NanoRepeat also supporting PacBio SMRT sequencing. However, many existing approaches remain challenging to implement in routine clinical settings owing to complex installation requirements and command-line-based workflows. In addition, these tools do not yet fully utilize all available information from the data, limiting their robustness and accuracy. These issues underscore the need for a comprehensive, user-friendly platform that simplifies the diagnostic workflow for repeat expansion disorders.

In this study, we present a diagnostic research platform for repeat expansion disorders that integrates Cas9 target enrichment, nanopore sequencing, and an automated analysis pipeline called RepeatLab. This platform delivers robust repeat-length estimation and features an accessible interface that requires minimal computing expertise. Multiplexing allows multiple samples (representing different patients or target genes) to be analyzed simultaneously, improving cost-effectiveness. Cas9-directed target enrichment enhances efficiency, and nanopore sequencing provides direct measurement of both repeat length and primary structure. The pipeline also reports repeated DNA sequences and DNA methylation states for each molecule.

We demonstrate the platform’s capabilities by measuring repeat profiles needed for diagnosis of DM1, a disorder caused by expansions of 50 or more repeats in the 3′ UTR of the *DMPK* gene. Using samples from 13 diagnosed patients, 4 unaffected controls, and 4 reference cell lines with long expansions, we evaluated repeat counts and sequence structures. We also examined hypermethylation near CCCTC-binding factor (CTCF) sites and the *SIX5* enhancer region at single-allele resolution, revealing its relationship to repeat length. Our findings show that multiplexed Cas9-based nanopore sequencing, coupled with the automated RepeatLab pipeline, provides the high-resolution repeat profiling at a reasonable cost and turnaround time, underscoring its potential for clinical applications.

## Materials and methods

### Ethics approval and consent to participate

This study is a retrospective study aimed at developing a diagnostic method, using anonymized residual samples after diagnosis has been completed. It has received an exemption from informed consent from the Samsung Medical Center Institutional Review Board (No. 2020-04-081).

### Consent for publication

The patient information was anonymized upon collection, ensuring that no personally identifiable information was retained. To identify individual cases, unique identification codes were used. This study was conducted with an exemption from informed consent.

### Samples and genomic DNA extraction

We used reference materials with known repeat numbers for expanded alleles from the Coriell Cell Repositories as follows: NA03991, 50–80 repeats; NA03697, 1.5 kb (up to 500 repeats); NA04034, 1.5–3.0 kb (up to 1000 repeats); NA03759, 4.5 kb (up to 2000 repeats). The number of repeats is known to be estimated using the Southern blot assay, except for NA03991, which has a short expansion. The determination of repeat numbers for the normal and small expansion (in NA03991) was carried out through RP-PCR method^18^.

We used clinical samples from patients who were subjected to *DMPK* gene analysis to confirm or exclude DM1. Genomic DNA was isolated from peripheral blood using MagNA Pure 96 DNA Isolation Kit (Roche) or Wizard Genomic DNA Purification Kit (Promega), following the manufacturer’s instructions.

### Repeat primed PCR and Southern blot

Conventional PCR was performed with fluorescent flanking primers as follows: DM1-F (5ʹ-FAM-GAAGGGTCCTTGTAGCCGGGAA-3′) and DM1-R (5′-GGAGGATGGAACACGGACGG-3′)^46^. RP-PCR was performed as previously described using primers as follows: P1-for (5′-FAM-GGGGCTCGAAGGGTCCTTGT-3′), P3 (5′-AGCGGATAACAATTTCACACAGGA-3′), and P4CAG-rev (5′-AGCGGATAACAATTTCACACAGGACAGCAGCAGCAGCAGCAG-3′)^18^. The largest number of repeats that can be estimated by PCR with fluorescent flanking primers and/or RP-PCR is 150 repeats^21,22^. For sizes larger than this, rough estimates of the number of repeats were made based on the characteristic saw-tooth patterns of the RP-PCR. For more direct measurements, Southern blots were performed on a clinical sample, DM151, as previously described^47,48^.

### CRISPR–Cas9 RNP-mediated DNA cleavage and library preparation

Cas9-based target enrichment experiments were performed with the ligation kit R9.4 version (SQK-LSK109/110, ONT) and according to the Cas9-targeted sequencing protocol (ENR_9084_v109_revD_04Dec2018, ONT). *DMPK* and *CNBP* targeting CRISPR RNA (crRNA) and *trans*-activating RNA (tracrRNA) were synthesized by Integrated DNA Technologies. The crRNAs were designed using CHOPCHOP^49^. crRNA and tracrRNA were pooled into an equimolar mixture with a total concentration of 100 μM. crRNA sequences were as follows: DMPK-1F 5′-AGTCCCCCACGTATATGGCAGGG-3′ and DMPK-1R 5′-CGGACAACCAGAACTTCGCCAGG-3′ or DMPK-R5 5′-GACGAGGTTACTTCAGACATGGG-3′ for *DMPK* gene region enrichment, and CNBP-F 5′-CGACAAAATACCTCTATCCG-3′ and CNBP-R 5′-TAGCGCTTTGGTGTAACTCATGG-3′ for *CNBP* gene region enrichment.

Target enrichment was performed on either *DMPK* alone or *DMPK/CNBP* dual loci. To generate RNP complexes, we mixed 10 µM annealed crRNA–tracrRNA pool and 62 µM Alt-R S.p. HiFi Cas9 Nuclease v3 (Integrated DNA Technologies) in 1× NEB CutSmart Buffer (New England Biolabs) and incubated at room temperature for 30 min. All steps were performed according to the manufacturer’s protocol for genomic DNA dephosphorylation, genomic DNA cleavage using Cas9-mediated RNPs, and target DNA dA-tailing. The 2–7 µg of genomic DNA was incubated with the RNPs for 20 min at 37 °C and then for 2 min at 80 °C to inactivate the enzyme. Sequencing adapters (AMX, ONT) were ligated to the dA-tailed target genomic DNA ends for 10 min at room temperature. One volume of tris-EDTA (pH 8.0) was added to the ligation mix to stop the reaction. Residual enzymes and salt were removed by adding 0.3× AMPure XP beads (Beckman-Coulter) followed by two washes with short fragment beads (SFB, ONT). The DNA library was eluted by incubation for 10 min at room temperature in elution buffer (EB, ONT).

### Library preparation of Cas9-targeted native barcoding

Cas9-multiplexing experiments of sample IDs ‘DM105’ and ‘DM112’ were performed with ligation sequencing kit (LSK-SQK109, ONT) and native barcoding expansion (EXP-NBD104/114, ONT) described as the manufacturer’s protocol for Cas9-targeted sequencing (Cas_native_v15, 2020, ONT). The steps for RNP preparation, dephosphorylation of genomic DNA, and cleavage of dA-tailed genomic DNA followed the manufacturer’s protocol in the same way as mentioned earlier. Native barcodes and dA-tailed genomic DNA ends were ligated together using Blunt/TA Ligase Master Mix (M0367, New England Biolabs). Equimolar amounts of each barcoded sample were pooled in a final volume of 65 µl of nuclease-free water. ONT sequencing adapters (AMXII from EXP-NBD104) were ligated, followed by purification, washing, and elution as described earlier for the singleplex experiments. The purified library was mixed with 37.5 µl of sequencing buffer and 25.5 µl of library loading beads.

### Nanopore sequencing

A total of five libraries were sequenced on Flongle flow cells (FLO-FLG001, ONT) with a MinION Mk1b device. In addition, a total of 20 libraries were sequenced on MinION flow cells (FLO-MIN106D, ONT) with a MinION Mk1b device (Supplementary Table 1). Each library was sequenced using MinKNOW 20.06.5 (ONT) until a plateau was reached.

### Comparative analysis of the effects of chunk size and model in basecalling of long repeats

Nanopore sequencing data were basecalled using Guppy v6.4.6 or Dorado v0.3.2 (ONT). The Guppy basecalling was performed exclusively with the ‘dna_r9.4.1_450bps_sup’ model using the command ‘guppy_basecaller --gpu_runners_per_device 2 --num_callers 2’. For Dorado, a number of newer models were evaluated, including ‘dna_r9.4.1_e8_fast@v3.4’, ‘dna_r9.4.1_e8_hac@v3.4’, ‘dna_r9.4.1_e8_sup@v3.3’, and ‘dna_r9.4.1_e8_sup@v3.6’, using the command ‘dorado basecaller --batchsize 128 --emit-sam’. The processing chunk sizes were either set to default (2000 for Guppy and 10,000 for Dorado) or increased to 80,000.

### Estimation of repeat counts using RepeatHMM, NanoRepeat, or STRique

To benchmark the accuracy of repeat count measurements using different programs, we prepared input data for each program according to its requirements and followed the respective guidelines. In the case of RepeatHMM^43^ and NanoRepeat^44^, raw FAST5 files were first basecalled using Guppy v6.4.6 or Dorado v0.3.2 (ONT). The resulting sequences were aligned to the GRCh38.p13 reference genome (GENCODE version 44) using the Minimap2 aligner^50^ with the settings ‘-x map-ont -k 13 -w 20’. For the analysis with RepeatHMM, we input the aligned BAM files with options ‘--MinSup 0 --SplitAndReAlign 2 --SeqTech Nanopore --outlog DEBUG’. Similarly, for NanoRepeat, we used the same BAM files and executed the program with ‘-t bam -d ont_sup’. To obtain repeat count values, we extracted them from the ‘p2sp’ lines in RepeatHMM’s result log file. For NanoRepeat, repeat counts were acquired from the phased read results that were specifically marked with ‘HIGH’ confidence. For STRique^36^, we used the ‘count’ command with the ‘r9_4_450bps.model’ file and the default settings. We then refined the results to include only those reads in which the prefix and suffix alignment scores were at least 4.

The benchmark data set for computational workloads comprised 1000 reads sampled from the DM98 sample. This data set included a targeted selection of 52 reads, each containing 100 or more repeat units from the *DMPK* locus and 948 random reads from non-repeat regions. We evaluated the running time for processing the data set by calling ‘time’ command. The measurements were repeated five times, and the average running time was calculated.

### Bootstrap analysis of coverage depth influence on repeat-length measurement

We randomly subsampled from the DM157 data set at 1× intervals from 1× to 50×, and 10× intervals from 50× to 280× coverage, with 5,000 replicates per coverage level. Repeat counts were called using both RepeatHMM and our modified method for each replicate. Percentiles (5, 20, 35, 50, 65, 80, and 95) of repeat counts were calculated for each allele at each coverage level. The minimum recommended coverage (12×) was determined as the lowest level where the 5th and 95th percentiles were within 20% deviation from the median repeat count.

### RepeatLab analysis

RepeatLab progresses through several automated stages, selecting specific target reads, repeat analysis, and methylation profiling. Initially, sketchy basecalling was performed on POD5 or FAST5 files using Dorado v0.3.2 (ONT), based on the dna_r9.4.1_e8_fast@v3.4 model. For multiplexed data, Dorado’s ‘demux’ command with ‘--barcode_kits EXP-NBD104’ was used for demultiplexing. The sequences from this basecalling step or provided in FASTQ format were mapped to the GRCh38.p13 human reference genome downloaded from GENCODE release 44 (ref. ^51^) using Dorado’s aligner command (options: ‘-k 20 -w 30 -I 6G’). Target sequence-mapped read IDs were extracted with SAMtools (command: ‘samtools view {bam file} {target region} | cut -f 1 | sort | uniq > read_ID_list’)^52^. The target region was determined as known repeat loci with 5000 bp of flanking sequences each for both upstream and downstream.

In the subsequent stage, extracted reads were re-basecalled for higher accuracy using Dorado and the dna_r9.4.1_e8_sup@v3.6 model, with adjustments for long repeats (‘--batchsize 128 --chunksize 80000’). These sequences were then mapped to the reference genome (options: ‘-k 13 -w 20 -s 40’). The quality of these results was assessed using Dorado summary and in-house scripts. Repeat counts were derived from the alignment files using RepeatHMM (options: ‘--MinSup 0 --SplitAndReAlign 2 --SeqTech Nanopore --outlog DEBUG’). Instead of RepeatHMM’s standard output, verbose debugging output was used to extract the unprocessed intermediate information. The repeat counts measured in the read level were log-transformed and fitted to a two-Gaussian mixture model, selecting the median of each cluster as the allele’s repeat length. The primary structures of repeat sequences were extracted using a modified version of RepeatHMM and visualized with alv^53^ to format the sequences in ANSI escape code.

Finally, the selected reads underwent another round of basecalling with a methylation-sensitive model using Dorado (‘--batchsize 128 --chunksize 80000 --modified-bases-models dna_r9.4.1_e8_sup@v3.3_5mCG@v0.1’). These sequences were mapped to the target chromosome sequence extracted from GRCh38.p13 (GENCODE release 44), using SAMtools and Dorado aligner. Methylation rates at each site were determined using the Modkit (ONT) pileup command with ‘--no-filtering --combine-strands --cpg’.

### Determination of differentially methylated region

We performed ordinary least-squares linear regression to examine the relationship between CTG repeat count and methylation rate. This was performed separately for each CpG site in clinical and cell line samples. Before running the regressions, we removed alleles with fewer than eight reads and CpG sites with insufficient coverage in more than five alleles. We then adjusted the *P*-values using the Benjamini–Hochberg method to control for multiple comparisons. CpG sites with an adjusted *P*-value less than 0.05 were considered differentially methylated.

### Running time measurement of RepeatLab

The processing performance of RepeatLab was evaluated using Google Colab Pro with a T4 GPU runtime in September 2023. All necessary files, including intermediate files, were managed directly through Google Drive mounts. Tests were repeated three times and average durations were recorded. The initial setup, including downloading the reference genome and software packages, was completed in advance and not included in the measurement of time.

## Results

### Overview of workflow

**Fig. 1:**
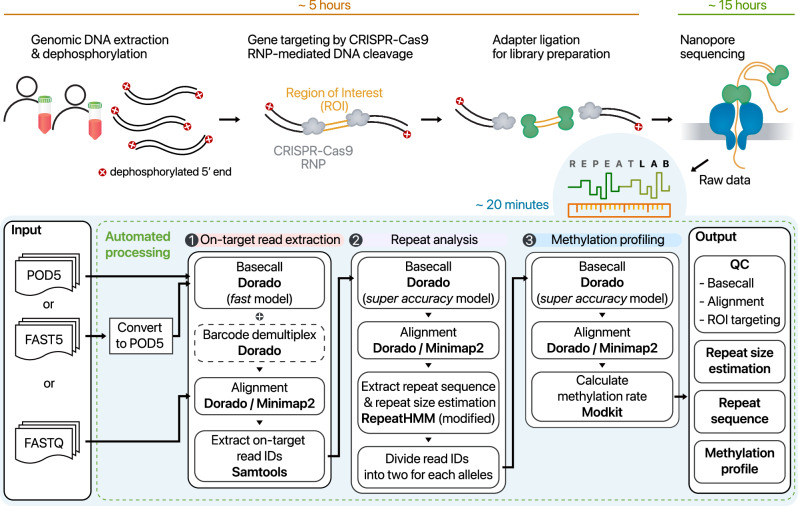
Workflow of the repeat expansion diagnostic analysis using CRISPR–Cas9 targeting and RepeatLab pipeline. Schematic diagram depicting the library preparation steps and the RepeatLab data analysis workflow including approximate time requirements for each step.

Our diagnostic workflow for repeat expansion involves four main stages: DNA extraction, library preparation, nanopore sequencing, and data analysis using RepeatLab (Fig. 1). To prepare the library, we adopted nanopore Cas9-targeted sequencing (nCATS) to the *DMPK* locus to enrich the target ROI^42^. The procedure begins with sample collection and extraction of genomic DNA, followed by dephosphorylation to render the existing DNA ends incompatible for adapter ligation. Subsequently, the desired gene region is targeted and enriched by Cas9-mediated DNA cleavage, which may simultaneously target multiple genes with different guide RNAs. After DNA cleavage, Cas9 RNP stays bound to the 5ʹ ends of the DNA, enabling preferential adapter ligation to the ROI. The sequencer then processes this adapter-ligated DNA, capturing the raw ionic current signals in POD5 or FAST5 format. RepeatLab takes over to perform basecalling, mapping, data quality checks, repeat size estimation, sequence structure analysis, and methylation profiling. The results are reported with organized figures and tables for easy interpretation. Generally, the entire process, starting from sample collection, is finished within a day (Fig. 1).

### RepeatHMM provides robust and fast measurement of repeat counts

To select a repeat counting tool for our pipeline, we compared three freely available tools for measuring repeat expansions from long-read sequencing data: RepeatHMM^43^, NanoRepeat^44^, and STRique^36^. RepeatHMM and NanoRepeat analyze sequenced reads as nucleotide sequences, whereas STRique processes nanopore raw signals. Our evaluation was carried out by running three tools on a total of 1445 on-target reads obtained from 20 samples sequenced for this study (Supplementary Table 1).

**Fig. 2:**
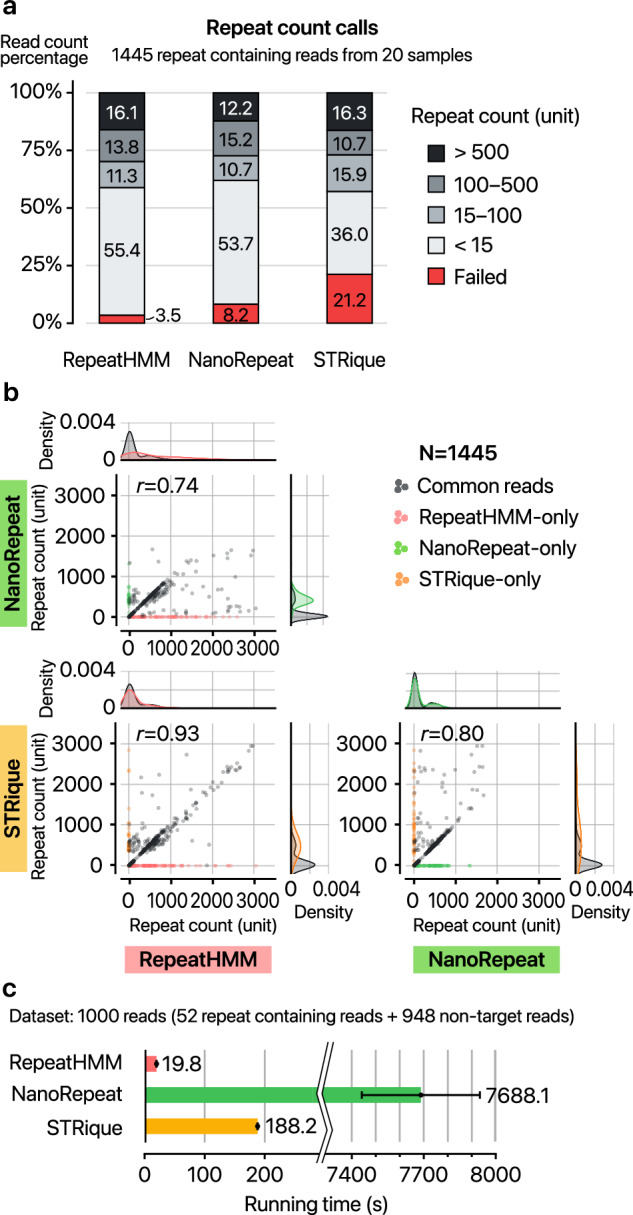
Comparison of repeat-length measurement tools for nanopore sequencing. **a** Distribution of repeat counts for all *DMPK*-mapped reads across the full data set, as determined by RepeatHMM, NanoRepeat, and STRique. **b** Pairwise comparisons of repeat count calls between any two of the three tools. Each dot in the scatter plot represents a single read, and the Pearson correlation coefficient (*r*) is shown in the upper-left corner. **c** Average running time for processing 1000 subsampled reads from clinical sample DM98 using each tool, with error bars indicating the standard deviation of three repeated runs.

Our results indicate that STRique failed to process 21.2% of the reads, whereas RepeatHMM and NanoRepeat showed substantially lower failure rates of 3.5% and 8.2%, respectively (Fig. 2a). Analysis of the failed reads revealed that most reads unprocessed by STRique were detected as short repeats by the other two tools (Fig. 2b). Beyond failure rates, we also observed notable discordances among successfully processed reads. Approximately 3.83% of reads identified as long repeats by STRique were classified as short by RepeatHMM. Additionally, NanoRepeat detected very short repeats in 4.3% of reads, whereas RepeatHMM and STRique consistently detected long repeats (Fig. 2b). Overall, RepeatHMM demonstrated the most robust performance in our test data sets, exhibiting both the lowest failure rate and the highest concordance with other tools when measurements were successful.

In terms of computational efficiency, RepeatHMM completed the analysis about 9.5 times faster than STRique and 388 times faster than NanoRepeat with a test data set consisting of 1000 reads (52 repeat containing reads and 948 non-target reads) (Fig. 2c). Memory usage for these programs was comparable. Given RepeatHMM’s superior robustness and computational efficiency, we integrated it into our RepeatLab pipeline.

### Modified strategy reliably detects repeat counts at low coverage down to 12×

**Fig. 3:**
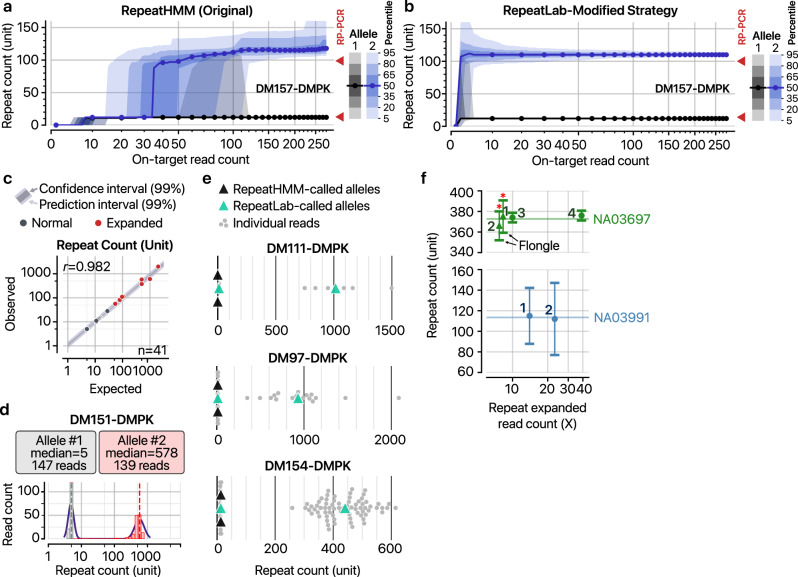
Benchmarks of RepeatLab’s modified strategy for repeat size estimation. Distribution of repeat counts determined by RepeatHMM (part **a**) or RepeatLab’s modified strategy at various on-target read depths (part **b**). For each read count, 5000 iterations of subsamples were generated from a single clinical sample. Lines and dots represent median repeat size estimates for the normal (black) and expanded (blue) alleles, respectively; shaded regions indicate percentile distributions as shown in the legend. Red triangles mark experimentally measured repeat sizes obtained via repeat primed PCR (RP-PCR). **c** Scatter plot comparing RepeatLab-estimated sizes with reference repeat sizes (measured by Southern blot or RP-PCR). Black dots indicate normal alleles and red dots indicate expanded alleles. The Pearson correlation coefficient is shown in the top-left corner. **d** Example of repeat size estimation by RepeatLab for sample DM151. Two allele clusters are displayed as a length histogram (bars) accompanied by the probability density function (line) from a fitted Gaussian mixture model. The vertical broken lines indicate the central value of each Gaussian distribution, corresponding to the normal (black) and expanded alleles (red). **e** Comparison of repeat size estimates generated by RepeatHMM and RepeatLab. Gray dots represent individual *DMPK* repeat reads, whereas triangles indicate the normal (left) and expanded (right) alleles per sample, shown in black for RepeatHMM and turquoise for RepeatLab. **f** Scatter plot illustrating consistency of repeat size measurements across different experimental setups or runs. Each dot represents the estimated size of an expanded allele from the indicated cell line samples, with error bars denoting the standard error of the mean (SEM) for multiple reads (*n* = 8, *n* = 8, *n* = 10, *n* = 39 for NA03697 in order; *n* = 14, *n* = 23 for NA03991 in order). Numbers near the dots correspond to the experiment identifiers in Supplementary Table 1 and Table 1. Red asterisks mark dots whose positions have been slightly shifted horizontally (±0.3) to improve readability.

To determine the minimum coverage required for accurate repeat count measurement, we subsampled on-target reads from DM157 data and evaluated RepeatHMM performance. At low read depths (<50 reads), the expanded allele calls became unstable, with a standard deviation of 47.0 at 30× (Fig. 3a). At intermediate depths (50–100×), the normal allele was instead mismeasured in ~20% of cases (Fig. 3a and Supplementary Fig. 1), as Gaussian centers became locked around the expanded allele, causing the algorithm to miss the shorter allele.

This limitation stems from RepeatHMM’s allele-representative calling algorithm, which fits linear-scale repeat counts to a Gaussian mixture model. The large variance of expanded allele values makes accurate center estimation difficult. To improve sensitivity at low coverage, we modified the calling strategy. Single-molecule repeat counts are first log-transformed, then clustered using a Gaussian mixture model (*n* = 2), with allele peaks determined by the median of each cluster. This approach yielded more accurate calls across all depths tested (Fig. 3b). For expanded alleles, median repeat counts remained stable down to ~10×, with mean relative absolute error below 0.08 at 12× (Supplementary Fig. 1). These results indicate that 12× represents the minimum coverage for reliable detection of expanded alleles with ~100 repeat units considering the accuracy requirements for the conventional clinical diagnostic applications.

We validated our approach by analyzing 50 alleles from 25 libraries and comparing results with Southern blot or repeat-primed PCR measurements (Table 1). Excluding nine alleles that could not be accurately measured by conventional methods, we observed strong correlation with previous measurements (Pearson’s *r* = 0.982; Fig. 3c). Our method accurately identified modal repeat lengths across broadly distributed measurements (Fig. 3d) and provided more robust estimates than RepeatHMM alone, even with only ~10 on-target reads (Fig. 3e). Results were highly reproducible, with low standard deviations across replicates (3.96 for NA03697 and 1.5 for NA03991; Fig. 3f). On the basis of these findings, we incorporated this modified strategy in RepeatLab.

**Table 1:** Repeat counts from reference assays and RepeatLab estimations.

Category		Sample ID		Known no. of repeats		RepeatLab measurement		
				Allele 1	Allele 2		Allele 1	Allele 2
**Normal**	**Clinical**	DM17		5	5		5	5
		DM115		5	12		5	12
		DM113		14	15		14	15
		DM112		13	28		13	28
**Abnormal**	**Reference**	NA03991	Rep-1	14	108		14	115
			Rep-2				14	112
		NA03697	Rep-1	12	500*		11	375
			Rep-2				12	366
			Rep-3				12	374
			Rep-4				12	376
		NA04034		12	1000*		12	605
		NA03759		14	2000*		14	1991
	**Clinical**	DM138		12	54		12	56
		DM153		13	79		12	82
		DM157		12	96		12	110
		DM01		12	>150		11	1047
		DM154		13	>150		13	441
		DM98		12	>150		12	459
		DM105		13	>150		13	501
		DM151		5	500*		5	578
		DM12		11	>150		10	429
		DM97		12	>150		12	932
		DM49		11	>150		11	1226
		DM110		5	>150		5	978
		DM111		11	>150		11	1015

Comparisons of the known repeat counts derived from reference assays — either Southern blot (asterisk (*)) or repeat primed PCR (RP-PCR) — with those obtained via the Cas9-enriched nanopore sequencing approach described in this study. ‘Rep’ indicates a technical or biological replicate.

### Multiplexing strategies enhance cost-effectiveness and scalability of repeat expansion detection

**Fig. 4:**
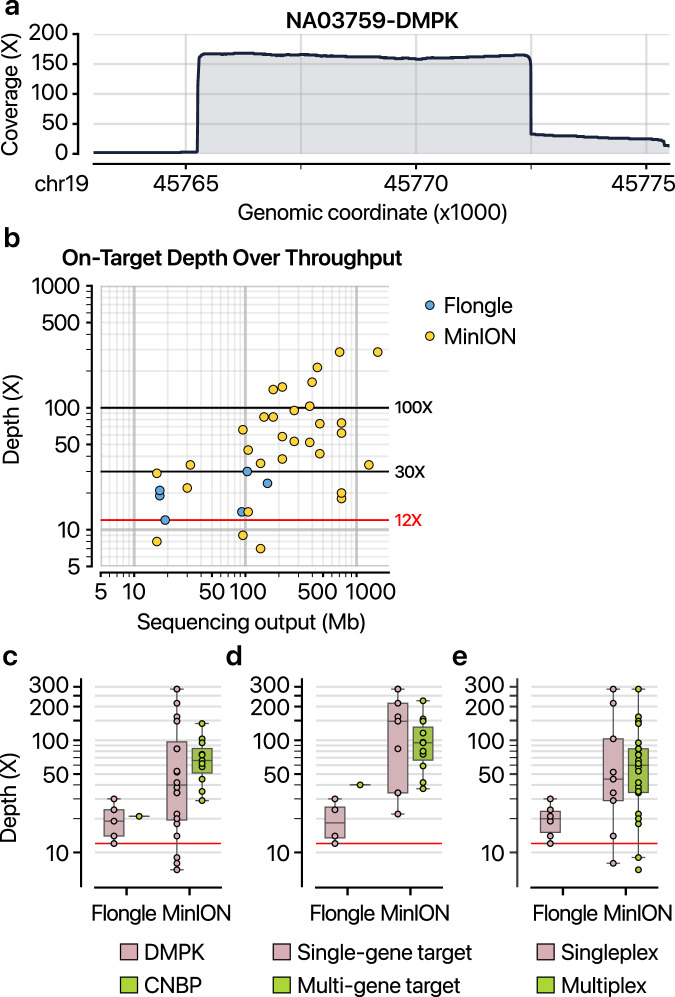
Efficiency of Cas9-targeted enrichment. **a** Coverage profile of sequenced bases around the *DMPK* target region (chromosome 19, coordinates 45,765,242–45,772,488) prepared from NA03759. **b** Total sequencing yield (*x*-axis) versus on-target coverage depth (*y*-axis) for runs performed on different flow cell types (Flongle or MinION). Each dot corresponds to a sequencing run of a particular sample. The red horizontal line marks 12× on-target coverage, identified in Fig. 3 as a confidence threshold for reproducible repeat size estimation. Box plots showing on-target coverage depth, comparing different target genes (part **c**), varying numbers of target genes within a single run (part **d**) and different sample multiplexing levels on Flongle or MinION flow cells (part **e**). The red horizontal line again indicates the 12~ coverage threshold.

We investigated multiplexing strategies using flow cells with different throughputs to improve the cost-effectiveness and scalability of repeat analysis. By pooling multiple samples or targeting multiple genetic regions, we sequenced 25 libraries in an average of 17.6 h (Supplementary Table 1). To test feasibility, we co-enriched the *CNBP* region, which undergoes expansion in myotonic dystrophy type 2 (refs. ^54,55^), alongside the *DMPK* gene. Cas9-mediated enrichment yielded 7–286× on-target depth for *DMPK* or *CNBP* regions. This enrichment was evident across libraries: the *DMPK* region in NA03759 showed ~160× coverage, far exceeding the ~0.12× genomic background (Fig. 4a). On average, we obtained 39× target gene coverage per 100 Mb of output (Fig. 4b). Under ideal conditions, a single MinION flow cell can analyze up to 100 patient genomes for repeat expansions using Cas9 target enrichment, although actual performance may vary.

We assessed the robustness of multiplex strategies by comparing on-target coverage across various experimental configurations. First, we evaluated whether enrichment efficiency varied by target gene. Both *DMPK* and *CNBP* showed comparable enrichment (19× and 21× for Flongle and 40× and 66× for MinION, respectively) (Fig. 4c). Importantly, 89% of samples achieved more than 12× on-target coverage, sufficient for individual-allele resolution of repeat expansions as discussed in the previous section. Next, we tested whether targeting multiple genes simultaneously would compromise performance. Multigene libraries performed similar to single-gene libraries (single-gene: 20× and 141× and multi-gene: 40× and 103×, for Flongle and MinION, respectively) (Fig. 4d), suggesting that diagnostic panels can be expanded without requiring additional sequencing throughput or compromising accuracy. Finally, we evaluated sample multiplexing through barcoding or sequential runs using wash-and-reuse protocols (Supplementary Table 1). These approaches also maintained the required on-target coverage (singleplex: 20× and 45× and multiplex: 60X, for Flongle and MinION, respectively) (Fig. 4e). In summary, Cas9-enriched nanopore sequencing with RepeatLab analysis offers flexible and scalable configurations suitable for diverse clinical and research applications while maintaining diagnostic accuracy.

### Basecalling optimization enables accurate detection of rare repeat interruptions

**Fig. 5:**
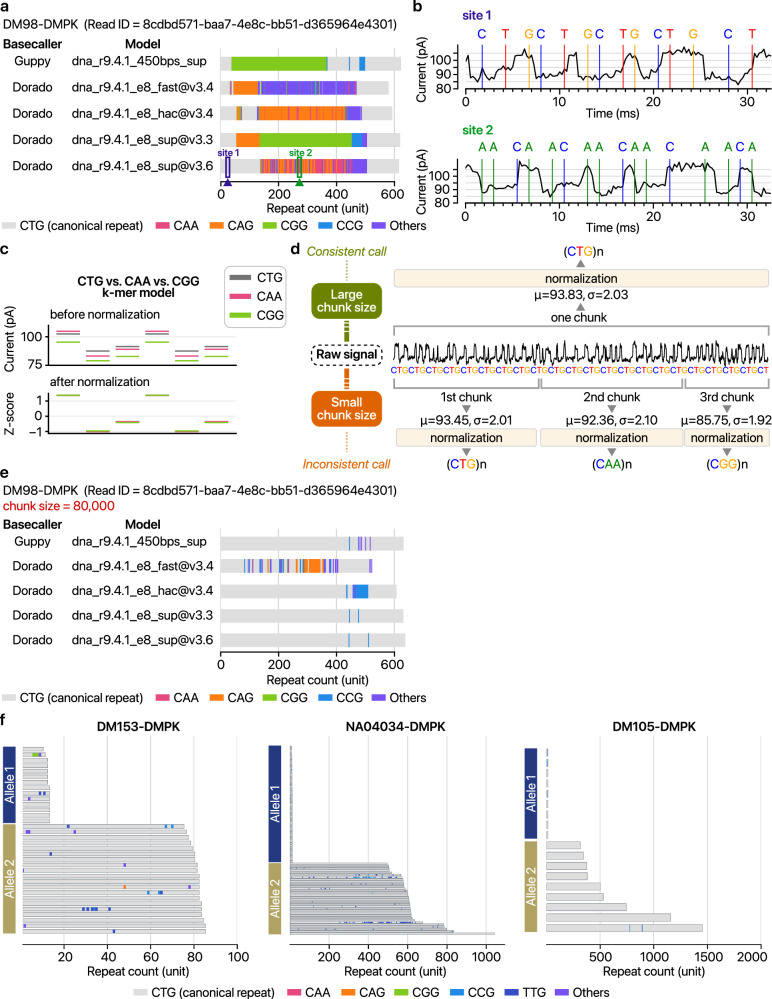
Optimization of basecaller parameters to reduce false-positive detection of repeat interruptions. **a** Comparison of repeat structures detected using different basecallers for the same example read. **b** Ionic current squiggle plots showing signal traces at two specific sites from panel **a**: site 1 (CTG repeat) and site 2 (CAA repeat). The base labels are positioned at the start of each *k*-mer event, reflecting the base at the center of that *k*-mer. **c** Signal trajectories before (top) and after (bottom) applying local scaling parameters. The top panel shows the ONT *k*-mer model, whereas the bottom panel demonstrates how local scaling affects the signal. **d** Schematic illustrating how small ‘chunk’ sizes in basecalling can cause ambiguity by deriving scaling parameters from segments that consist exclusively of repetitive sequences. *µ*, mean ionic current; *σ*, standard deviation. **e** Repeat structures from the same example read shown in panel **a**, basecalled with an increased chunk size (80,000). All other parameters remained the same. **f** Detailed map of repeat interruptions spanning full repeat sequences in patient sample DM153, reference cell line NA04034 and sample DM105. Gray bars denote CTG repeats, whereas colored segments indicate alternative sequences (for example, CAA), as specified in the legend.

We investigated the detection of repeat interruptions, specifically the non-CTG sequences occurring within the CTG repeats^56–61^. These interruptions, found in 3–5% of cases, are linked to milder symptoms or late onset of repeat expansion disorders^58,60–63^. Our preliminary analysis identified notable CGG and CAG interruptions in the 3′ UTR region of the DMPK gene (Fig. 5a), confirming previous findings^64^. However, we discovered that the types and locations of these interruptions varied considerably depending on the basecallers and models used (Fig. 5a). For example, in a read from patient DM98, two sites marked in Fig. 5a were identified as CTG and CAA repeat, respectively, but their raw ionic current signals were nearly identical (Fig. 5a,b). We hypothesized these variations might be due to basecall errors caused by improper normalization of long homogeneous sequences (Fig. 5c). To address this issue, we increased the basecalling chunk size to include the entire span of a long repeat with sufficient flanking non-repeat sequences (Fig. 5d). This adjustment significantly reduced apparent repeat interruptions, converting most back into canonical CTG stretches (Fig. 5e). More thorough basecalling configurations reported fewer interruptions, suggesting that many were artifacts of algorithmic limitations. On the basis of these findings, RepeatLab adopts the ‘super-accuracy’ basecalling model with larger processing chunks to minimize erroneous identification of repeat interruptions, ensuring a more precise diagnosis.

Contrary to previous findings using nanopore sequencing that repeat interruptions primarily occur in the middle of long repeats^64^, our data show an even distribution of these interruptions across the entire repeat region (Fig. 5f). Despite the examination of 74 alleles, no statistically significant repeat interruptions were identified, with only inconsistent types and locations reported within individual alleles (Fig. 5f). By enhancements in basecalling that reduce the misidentification of interruptions, RepeatLab offers improved sensitivity for detecting rare repeat interruptions for further research, characterizing the correlation between genotypes and pathogenicity in repeat expansion disorders^58,60,61,65–67^.

### Comprehensive methylation profiling provides a basis for investigating DM1 pathogenesis beyond repeat size

**Fig. 6:**
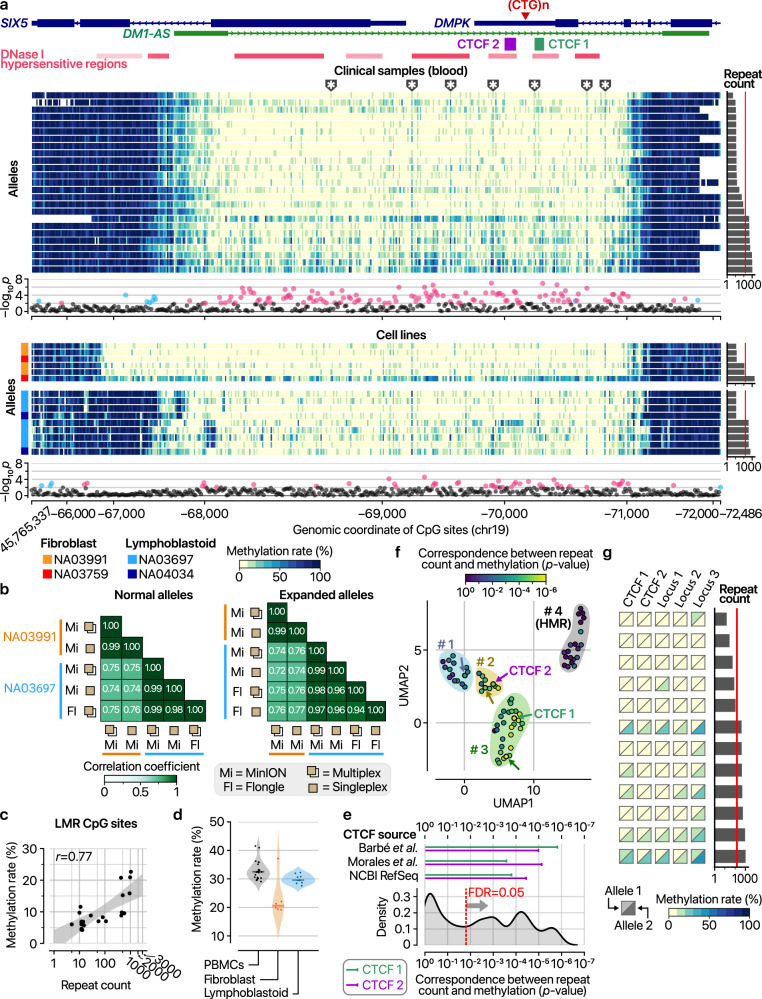
Methylation profiles near the *DMPK* repeat region. **a** Methylation profiles of alleles from both clinical (top) and cell line (bottom) samples in the neighborhood of the *DMPK* repeat region. Each column in the heatmap represents a CpG site, and each row corresponds to a specific allele. Gray bars on the right show the estimated repeat count for each allele, with a red vertical line marking 150 repeats. Below the heatmap, scatter plots illustrate the correlation between methylation and repeat count (*P*-value from ordinary least squares). CpG sites with a Benjamini–Hochberg false discovery rate (FDR) under 0.05 are shaded pink for positive slopes or sky blue for negative slopes. Gene annotations are based on GENCODE v47. CTCF binding sites are indicated according to ref. ^73^, and DNase I hypersensitive regions are represented by varying opacity (ENCODE v4). **b** Heatmaps showing Pearson correlation coefficients for methylation profiles of replicate samples sequenced under different conditions (MinION versus Flongle flow cells, singleplex versus multiplex runs). Methylation rates were calculated for bins averaging six consecutive CpG sites in the target region to reduce noise and facilitate comparison. **c** Scatter plot illustrating the relationship between repeat counts and methylation rates within the lowly methylated region (LMR) (chr19:45,767,872–45,770,996 in GRCh38). Each dot represents a single allele with more than eight supporting reads. The Pearson correlation coefficient is displayed in the top-left corner, and the 95% confidence interval is shown as a gray shaded area. **d** Violin plots displaying the distributions of average methylation rates within the LMR, grouped by cell type. Each violin shows the kernel density estimates of observed values, and the central horizontal line denotes the median. **e** Distribution of *P*-values for associations between methylation rates and repeat counts across all CpG sites, assessed by ordinary least-squares regression. The red dashed line indicates FDR = 0.05. Horizontal bars in the top panel highlight significance in CTCF-bound regions alternatively annotated by the previous studies^73,81,82^. **f** UMAP visualization of all CpG loci around the *DMPK* repeat, highlighting the strength of the methylation–repeat count association. Each point represents a group of six consecutive CpG sites and is colored by the *P*-value of the ordinary least-squares regression between methylation rate and repeat count. Color-shaded clusters group similar loci, and an arrow marks the locus with the lowest *P*-value within each cluster. **g** Methylation profiles of selected loci from each of two alleles in patient blood samples. Each row corresponds to a patient sample, with the expanded allele’s *DMPK* repeat count indicated on the right. The red line on the bar plot marks 150 repeats. PBMC, peripheral blood mononuclear cell.

Methylation status of the mutant allele better predicts DM1 clinical phenotype compared with repeat expansion size measured from a patient’s blood^12,68–80^. Thus, we extended RepeatLab to report summarization of methylation status alongside repeat length. First, we investigated the haplotype-level methylation profiles in our samples with >12 reads covering the majority of the region (Fig. 6a and Supplementary Tables 2 and 3). Consistent with previous findings^64,72^, regions neighboring the repeats in both the *DMPK* and *SIX5* genes were nearly completely methylated across individuals, alleles, and cell types (Fig. 6a). However, in the intergenic region — from the first intron of *SIX5* to the penultimate intron of *DMPK*— the methylation levels dropped below 10%, irrespective of repeat length (Fig. 6a).

Methylation levels were elevated at certain sites within the lowly methylated region (LMR) in both expanded and non-expanded alleles (Fig. 6a). Methylation profiles were reproducible across repeated analyses of the same sample, comparisons between singleplex and multiplex analyses, and across different sequencing devices (Fig. 6b). Remarkably, hypermethylation within the LMR was more pronounced in alleles with longer repeat expansions (Fig. 6c). Of note, fibroblasts and lymphoblastoid cell lines remained with generally lower methylation levels than peripheral blood mononuclear cells for haplotypes with similar repeat expansions (Fig. 6d).

To identify regions exhibiting repeat-associated differential methylation, we evaluated the correlation between repeat expansion size and methylation levels at the haplotype level using robust linear regression in both peripheral blood mononuclear cells and other cell lines (Fig. 6a). Differentially methylated sites were dispersed throughout the LMR without significant clustering in specific regions. Most CpG sites in DNase I hypersensitive regions exhibited the highest methylation levels within the LMR across all repeat-expanded alleles but remained largely unmethylated in normal alleles (Fig. 6a). Consistent with previous findings^73,81^, the upstream CTCF binding site (CTCF 1) and downstream CTCF binding site (CTCF 2) showed elevated methylation levels relative to surrounding regions and a strong correlation with repeat expansion size (Fig. 6e).

On the basis of our methylation profiling, we identified five information-rich regions for summarized reporting in RepeatLab (Fig. 6f). These include CTCF 1 and CTCF 2, previously reported to be highly associated with DM1 phenotypic manifestations^73,81^, as well as three loci that constitute the most significant regions among four groups classified by similar methylation profile patterns (Supplementary Fig. 2 and Supplementary Table 4). In our clinical samples, the methylation rate of allele 2 in locus 3 consistently correlated with repeat count (Fig. 6g). However, the methylation status of CTCF 1, CTCF 2, locus 1, and locus 2 individually was not sufficient to infer the repeat count (Fig. 6g). RepeatLab automatically processes these methylation analyses and provides quantification of methylation levels for the key loci. Further investigation into these five loci, in combination with clinical phenotypic labels, may facilitate the development of robust criteria for DM1 prognosis and subtyping.

### RepeatLab analysis finishes within an hour using a public computational environment

**Fig. 7:**
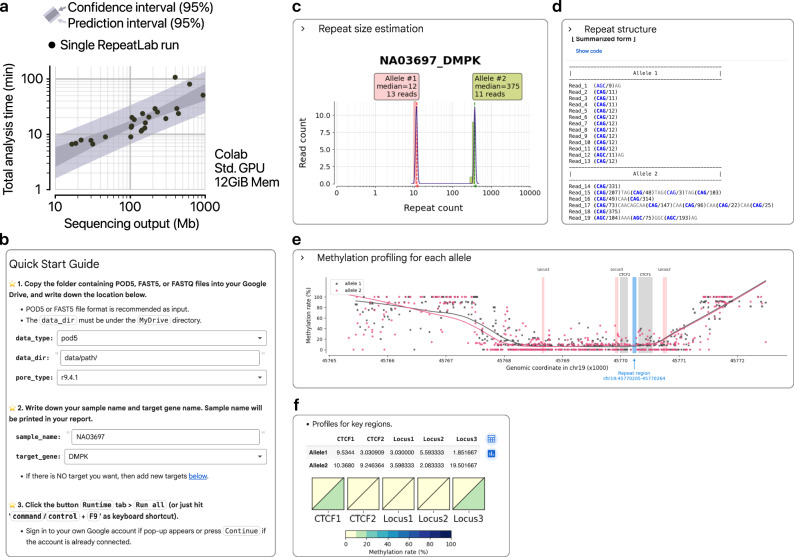
Benchmark and user interface of RepeatLab. **a** Scatter plot illustrating the total time (*y*-axis) required for RepeatLab analysis of each sequencing run, plotted against the total sequencing throughput (*x*-axis), when executed in a standard Google Colab environment. Each point represents the average of three repeats per run. The darker shading denotes the 95% confidence interval, whereas the lighter shading indicates the 95% prediction interval, reflecting possible variability in future data sets. **b** Screenshot of the RepeatLab analysis setup form, highlighting key input parameters, configuration options, and file selection fields for automated runs. Representative snapshots of the RepeatLab output interface. **c** Summary of repeat count calls, displaying detected repeat lengths across all alleles or samples to provide an immediate overview of expansions. **d** Visual representation of repeat structures, including summarization of any detected interruptions. **e** Methylation profiles across the target repeat region for detailed inspection of local methylation patterns near the target region. **f** Summarized methylation rates at user-defined key loci (as described in Fig. 6), enabling quick and routine interpretation for each analyzed sample.

To assess the computational efficiency of RepeatLab, we evaluated its performance both in a publicly available environment (Google Colab) and on a local machine. When configured with default parameters and a standard GPU option, a Google Colab virtual machine equipped with 12 GiB RAM was capable of analyzing up to 1000 Mb of sequencing data in ~80 min (Fig. 7a). These findings demonstrate that RepeatLab can handle medium-to-large-scale data sets in clinical research settings without requiring a dedicated computational environment. Furthermore, the software processed 100 Mb of sequencing data — which is sufficient for targeted analysis of a single repeat locus — in around 10 min, indicating its potential to deliver rapid results with minimal computational resources (Fig. 7a). For large sample cohorts, RepeatLab can also be deployed on a Linux workstation or server, which provides additional scalability as throughput increases. The runtime appeared to increase linearly with sequencing throughput (Fig. 7a), allowing computing equipment requirements to scale proportionally with the desired throughput.

We designed RepeatLab to prioritize ease of adoption, requiring minimal user input to make it widely accessible. Users need to only provide the path to input files and the target gene of interest (Fig. 7b). Key tasks such as generating sequencing quality metrics, identifying repeat counts (Fig. 7c), analyzing potential repeat structures (Fig. 7d), and profiling methylation near the target region (Fig. 7e, f) are fully automated. Users can initiate the analysis by entering sample and data format details in a single Google Colab notebook, in which all results — including quality metrics, repeat count estimations, and methylation analyses — are presented clearly. RepeatLab is designed to facilitate the clinical research in rapid and information-rich detection of repeat expansions for prognosis and genetic counseling, providing a resource-efficient solution for researchers and clinicians.

## Discussion

This study demonstrates a new diagnostic approach for repeat expansion disorders by combining Cas9-targeted nanopore sequencing with the streamlined RepeatLab analysis pipeline. In patient samples with DM1, the platform simultaneously assesses repeat expansion size, interruption profiling, and methylation status at the individual allele level, offering a comprehensive understanding of the mutational landscape.

A key strength of this approach lies in its improved accuracy for measuring repeat lengths, even at relatively low sequencing coverage. By revising the repeat count calling strategy, the pipeline produces stable and consistent estimates of expanded allele lengths, despite limited on-target reads. This feature is particularly beneficial for multiplexed designs, in which lower sequencing depth can be cost-effective without sacrificing precision.

The optimized basecalling strategy further refines the process, enabling rapid data processing while accurately detecting both repeat lengths and potential interruptions. A two-pass basecalling workflow — incorporating an initial draft analysis followed by a high-quality, restricted second pass — balances efficiency and accuracy. In addition, extending the signal window size in basecalling reduces most false-positive interruption calls. This improved specificity is crucial for investigating the clinical importance of interruptions and their potential relationship to disease progression. Applying RepeatLab to a broader set of clinical samples could clarify this relationship and help refine phenotype predictions.

RepeatLab also streamlines methylation profiling surrounding the expanded repeat, providing allele-specific methylation information that may better predict DM1 disease severity compared with expansion size alone. In turn, this enhanced characterization of epigenetic status could aid in establishing more robust prognostic criteria or defining subtypes in DM1.

Despite these advantages, several limitations must be acknowledged. First, the methylation profiling module relies exclusively on nanopore sequencing technology, thereby excluding PacBio platforms. Second, accurate repeat analysis requires capturing sufficient flanking sequences, which complicates the analysis of very long expansions (>50 kbp). Finally, the discontinuation of ONT’s Cas9-targeted sequencing kit and R9.4 flow cells necessitates the use of third-party reagents and introduces additional technical steps for implementing the current V14 chemistry.

Future developments of RepeatLab could involve evaluating a broader range of clinically diverse sample sets, including those representing different disease subtypes, to further enhance its utility and comprehensiveness in clinical applications. Overall, the platform’s scalability, user-friendly design and comprehensive features make it a valuable resource for both research and clinical use, contributing to deeper insights into the molecular underpinnings and genotype–phenotype correlations of repeat expansion disorders.

## Supplementary information

Supplementary Information

Supplementary Tables

## Data Availability

Due to patient privacy concerns, the raw sequencing data supporting the findings of this study are not publicly available but are available upon reasonable request from the corresponding author. Samsung Medical Center stores data with restricted access.

## Code availability

RepeatLab can be accessed at https://colab.research.google.com/github/ChangLabSNU/RepeatLab/blob/main/RepeatLab.ipynb. Analysis pipeline codes written in Snakemake and manual for Linux command line interface can be accessed at https://github.com/ChangLabSNU/RepeatLab.
