# Supplementary material for: Targeted long-read sequencing for high-resolution repeat profiling in myotonic dystrophy type 1
Source: Exp Mol Med. 2026 Apr 13;58(4):1203–15. doi: 10.1038/s12276-026-01683-6 (PMC13144457; doi:10.1038/s12276-026-01683-6)
Supplement: Supplementary file 1 — Supplementary Information [file 12276_2026_1683_MOESM1_ESM.pdf]

Supplementary Information

Targeted Long-Read Sequencing for High-Resolution Repeat Profiling in Myotonic Dystrophy Type 1

Yoojung Han, Ja-Hyun Jang, and Hyeshik Chang

Supplementary Fig. 1. Repeat count estimation precision across different sequencing coverages

Supplementary Fig. 2. Association between methylation rate and repeat length across the *SIX5-DMPK* lowly methylated region (LMR)

(The supplementary tables have been provided as a separate Excel file.)

Supplementary Table 1. Summary of nanopore sequencing runs and corresponding on-target depths

Supplementary Table 2. Methylation rates of individual alleles in clinical samples

Supplementary Table 3. Methylation rates of individual alleles in cell line samples

Supplementary Table 4. Subregions within the *SIX5-DMPK* lowly methylated region (LMR) and their methylation profiles

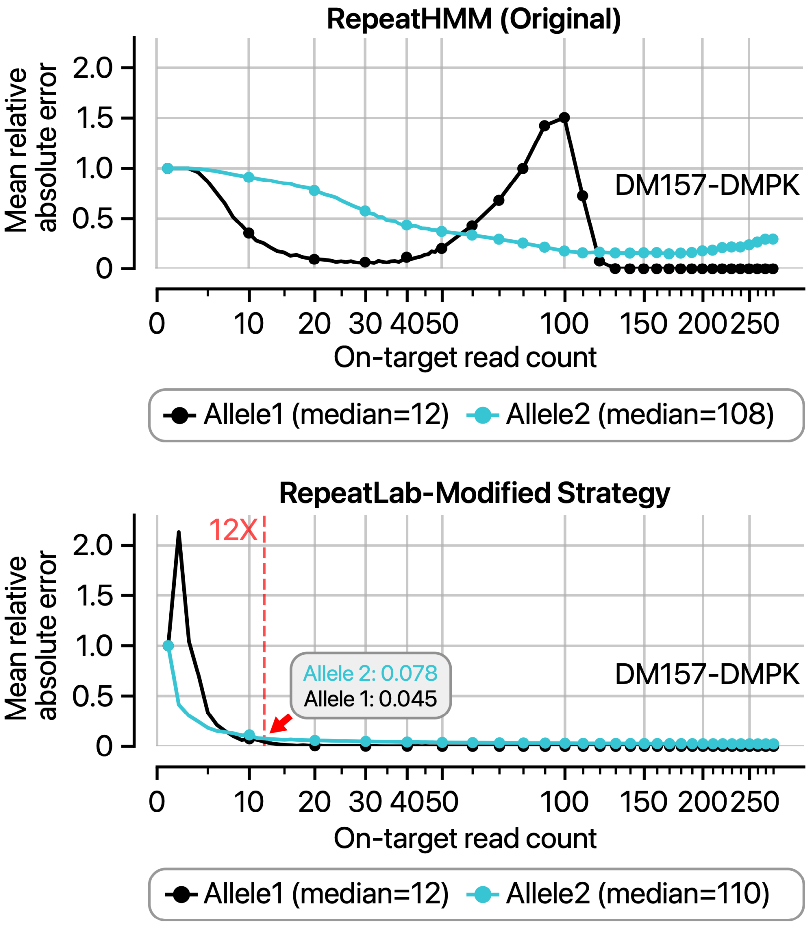

Supplementary Fig. 1. Repeat count estimation precision across different sequencing coverages

Mean relative absolute error (RAE) of repeat counts estimated by RepeatHMM or RepeatLab at various on-target read depths. RAE was calculated as |subsampled estimate – reference median| / reference median, where the reference median was derived from all reads. Bootstrap subsampling was performed 5000 times at each read depth. Black and turquoise lines represent allele 1 (reference median = 12 repeats for both methods) and allele 2 (reference median = 108 repeats for RepeatHMM; 110 repeats for RepeatLab), respectively.

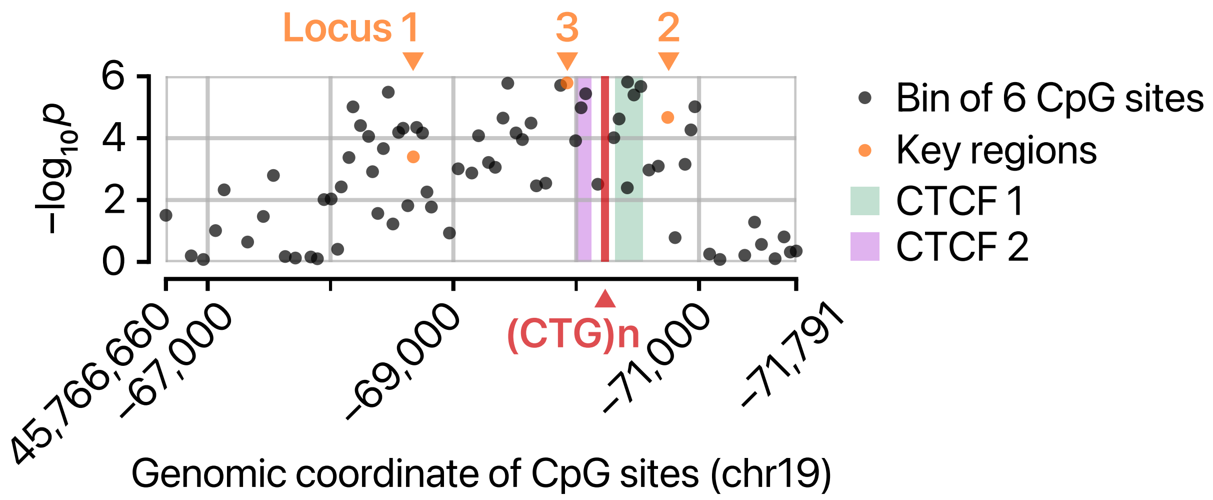

Supplementary Fig. 2. Association between methylation rate and repeat length across the *SIX5-DMPK* lowly methylated region (LMR)

Scatter plot showing the significance of the association between methylation rate and repeat count, based on an ordinary least squares regression analysis. Each dot represents a subregion of six consecutive CpG loci. Representative loci from each cluster (Fig. 6g) are highlighted in yellow. Two CTCF binding sites defined in Barbé *et al.* [1] are marked in color.

(The supplementary tables have been provided as a separate Excel file.)

Supplementary Table 1. Summary of nanopore sequencing runs and corresponding on-target depths

List of all nanopore sequencing runs performed in the study and the resulting on-target coverage depths for each sample. For multiplexed runs, the flow cell was used twice (labeled “1st” and “2nd”) with an intermediate wash step. Runs labeled “Barcoded” include the barcoded samples DM105 and DM112.

Supplementary Table 2. Methylation rates of individual alleles in clinical samples

List of methylation rates of individual alleles at CpG sites near *DMPK* and *SIX5* (chr19:45,765,337–45,772,486, GRCh38) in clinical samples. Samples DM49, DM115, and DM111 were excluded due to a DMPK on-target sequencing depth of less than 12X (refer to Supplementary Table 1). Additionally, alleles with sequencing coverage below 8—specifically, allele 1 of DM110 and alleles 1 and 2 of DM17—were also excluded. Positions with no aligned read are left blank.

Supplementary Table 3. Methylation rates of individual alleles in cell line samples

List of methylation rates of individual alleles at CpG sites near *DMPK* and *SIX5* (chr19:45,765,337–45,772,486, GRCh38) in cell line samples. The only excluded allele was allele 1 of the NA03697 Flongle singleplex sample, which had only four on-target reads.

Supplementary Table 4. Subregions within the *SIX5-DMPK* lowly methylated region (LMR) and their methylation profiles

List of all subregions in the *SIX5-DMPK* lowly methylated region, each comprising six consecutive CpG sites. Five key subregions (loci) are labeled in the “Key Locus Name” column, and their associated cluster numbers (Fig. 6g) appear under “Cluster.” The “P-value” and “Regression Slope” columns report ordinary least-squares results for the association between repeat count and methylation rate at the allele level. The “Average Methylation Rate” column shows the mean methylation rate of the six CpG sites across all samples.

Reference

1 Barbé, L. *et al.* CpG methylation, a parent-of-origin effect for maternal-biased transmission of congenital myotonic dystrophy. *Am J Hum Genet* **100**, 488-505 (2017).
